# Dosimetric impact of reduced nozzle-to-isocenter distance in intensity-modulated proton therapy of intracranial tumors in combined proton-carbon fixed-nozzle treatment facilities

**DOI:** 10.1186/1748-717X-8-218

**Published:** 2013-09-18

**Authors:** Urszula Jelen, Marta E Bubula, Filippo Ammazzalorso, Rita Engenhart-Cabillic, Uli Weber, Andrea Wittig

**Affiliations:** 1 Department of Radiotherapy and Radiation Oncology, Philipps-University of Marburg, Marburg, Baldingerstrasse 35043, Germany; 2 Particle Therapy Center, Rhön Klinikum AG, Marburg 35043, Germany

**Keywords:** Intensity-modulated proton therapy, Pencil beam, Raster scanning, Brain tumor, Normal tissue sparing, Treatment room geometry

## Abstract

**Background:**

In combined proton-carbon fixed-nozzle treatment facilities with raster scanning delivery, the scattering of proton pencil beams caused by nozzle elements and the relatively large nozzle-to-isocenter distance cause a beam broadening. This may pose limitations to the achievable dose conformity. One way to counteract this effect is by delivering the treatment in a position closer to the nozzle than the room isocenter. Purpose of this study was to assess the potential dosimetric benefit of such solution, in terms of dose conformity and normal tissue sparing, in intensity-modulated proton therapy (IMPT) of intracranial tumors.

**Material and methods:**

For 12 patients with intracranial lesions, IMPT-plans were created at two treatment positions: nozzle-to-treatment-isocenter distance: 100 cm (room isocenter) and nozzle-to-treatment-isocenter distance: 60 cm. The resulting plans were compared in terms of dose distributions, dose-volume histograms and selected dosimetric indexes.

**Results:**

With comparable target coverage, statistically significant normal tissue sparing was achieved through the reduction of the distance between nozzle and treatment isocenter. The decrease in mean dose (D_mean_) was 12.5% to the whole brain, 16.2% to the brainstem, 9.7% and 15.4% to the temporal lobes, 10.0% and 12.9% to the hippocampi, 11.8% and 12.5% to the optic nerves and 0.2% to the chiasm. The volume receiving at least 10% of the prescribed dose (V_10%_) was reduced by more than 10% for most organs at risk (OARs). The maximum dose (D_near-max_) values to most OARs remained without significant difference.

**Conclusion:**

A reduced distance between nozzle and treatment isocenter leads to steeper lateral dose gradients and significantly reduces the volume of OARs adjacent to the target, which receives low to intermediate doses. Technical solutions shifting the treatment isocenter closer to the nozzle should be considered in clinical situations, where critical OARs are adjacent to the beam channel and where the integral dose should be minimized.

## Background

High precision radiotherapy is especially valuable in the treatment of intracranial tumors, due to the direct vicinity of critical structures. The finite range and precise high-dose deposition towards range end (Bragg peak) make proton beam therapy favorable for such treatments, where a high degree of conformity is required [1,2]. Although to date only a few clinical studies comparing protons to high-energy photons have been conducted, dosimetric comparisons of treatment plans demonstrated a reduced involvement of normal tissues [3-5]. Hence it is expected, that the advantageous proton dose distributions will result in reduced normal tissue morbidity.

In particular, application of the raster scanning technology, as opposed to passive beam scattering, offers further potential advantage in terms of improved dose conformity. A factor determining the achievable conformation is the size of the available beam, as smaller beams allow forming steeper dose gradients. However, when using raster scanning, proton beams undergo an unavoidable spread, induced by multiple Coulomb scattering and nuclear interactions within the beam delivery and monitoring systems of the treatment nozzle. This effect is much more pronounced for protons as compared to heavier ion species used in radiotherapy (e.g. carbon ions) and results in marked beam broadening [6,7]. In order to limit further degradation of the lateral penumbra, due to the geometrical beam divergence, the distance between the nozzle and the patient should be kept as short as possible.

However, in combined proton-carbon fixed-nozzle raster-scanning treatment facilities, the geometry of the treatment rooms typically represents a trade-off between technical solutions in beam delivery, flexibility of patient positioning and therapeutic requirements (e.g. degrees of freedom of room positioning and imaging robotic devices), resulting, in the example of our facility, in a nozzle-to-isocenter distance of 100 cm. This leads to a reduction of the pencil beam sharpness at the room isocenter, possibly limiting the achievable dose distribution conformity.

**Figure 1:**
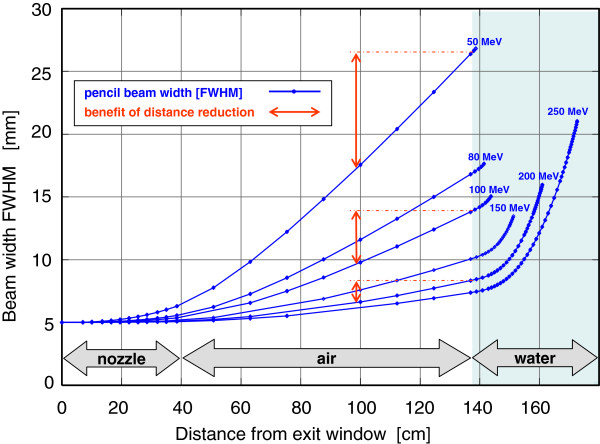
**Calculated spread of a proton beam from a nozzle geometry compatible with the ion beam therapy setup at the GSI**[8,9]**and with recent Siemens combined ion therapy facilities, like the one at University Medical Center of Marburg.** An initially parallel particle beam enters a water absorber (patient) at 1 m distance from the nozzle exit. If the water absorber is shifted closer to the nozzle, the width of the beam entering the water is reduced. The resulting difference in beam width in the patient for a 40 cm shift is explicitly marked (red line) at three exemplary energies (50, 100 and 200 MeV).

To minimize this problem, at our particle therapy facility, application of the Extended Penumbra Reduction (EPR) technology (Siemens AG, Healthcare Sector, Erlangen, Germany) has been planned. This technology, integrated in the planning and delivery systems, enables patient treatment at reference points that are closer to the nozzle than the room isocenter and reduces the proton pencil beam full-width-at-half-maximum (FWHM) in the target by approximately 2–8 mm (see Figure 1) [8,9].

Since the greatest dosimetric advantages occur for lower energies of the incident proton beams (see Figure 1), clinical benefits are to be expected in the case of rather superficially located tumors adjacent to critical structures, where tumor control and normal tissue sparing are both primary and uncompromising objectives. A typical situation, where such highly conformal dose distribution is desired, is the treatment of pediatric and/or benign intracranial tumors.

The purpose of this study was to assess the potential dosimetric benefit of a solution, where the patient is shifted towards the nozzle for treatment, in terms of the dose conformity and normal tissue sparing in intensity-modulated proton therapy (IMPT) of intracranial tumors, in the context of the specific geometric limitations of a fixed-nozzle facility.

## Methods

### Patient data

Twelve patients with intracranial tumors treated at the Department of Radiotherapy and Radiation Oncology of the University Medical Center of Marburg with photon stereotactic radiotherapy (SRT) between 2003 and 2011 were selected for this planning study. Patient characteristics are summarized in Table 1.

**Table 1:** Patient characteristics

**Number of patients**	**12**
Gender	
male/female	3/9
Age (y)	
median	14
range	3-76
Tumor type	
germinoma	1
recurrent medulloblastoma	1
optic glioma	2
ependymoma	1
pilocytic astrocytoma	2
meningioma	5

Written informed consent was obtained from the patient for the publication of this report and any accompanying images.

### Imaging and treatment planning

Computed tomography (CT) and magnetic resonance (MR) images were acquired for all patients in the supine position. Image fusion guaranteed for precise target and normal tissues delineation. The gross tumor volume (GTV) was identified as the macroscopic tumor mass and the planning target volume (PTV) was created by adding a 1 mm 3D-uniform margin to the GTV [10]. The median PTV volume was 54.8 cm^3^ (range: 11.2-147.0 cm^3^). The whole brain, brainstem, temporal lobes, hippocampi, optic chiasm and optic nerves were outlined. The brain, brainstem, optic chiasm, optic nerves as well as the parts of temporal lobes and hippocampi not overlapping with the PTV were defined as organs at risk (OAR).

For the sake of result comparison within the study, a common prescription was assigned to all cases, with a total dose of 50.4 Gy(RBE) in 28 fractions to the PTV, using a constant relative biologic effectiveness (RBE) factor of 1.1, to convert between absorbed dose [Gy] and biologically weighted dose [Gy(RBE)] [11]. The planning objective was to deliver more than 95% of the prescribed dose to at least 95% of the PTV. Dose constraints to the OARs were specified according to available guidelines [12].

The treatment plans were generated with syngo^®^ PT Planning VA11 (Siemens AG, Healthcare Sector, Erlangen, Germany). The beam directions were chosen individually for each patient to avoid unnecessary dose, especially to the optic nerves and hippocampi. For three patients, a lateral parallel-opposed beam configuration was found satisfactory from a clinical point of view, while for the remaining nine patients, isocentric couch rotations were employed to achieve more favorable setups with oblique cranial beams. For each patient, two plans were created, adopting two different patient positions, defined by the distance between nozzle and the treatment isocenter: 100 cm, placing the treatment isocenter in coincidence to the room isocenter, and 60 cm, shifting the treatment isocenter 40 cm towards the nozzle and away from the room isocenter. These two distances are referred to, in the remainder of the text, as d_ISO_ and d_ISO-40_, respectively. Both treatment isocenter positions (and all intermediate positions) can currently be technically realized at our particle therapy facility.

The treatment planning system used for this study contains a set of tabulated measured spot sizes in air, recorded during commissioning and yielding a parameterization of the envelope of the scattered proton beam. Hence spot sizes that can be requested during treatment planning, correctly model effects of nozzle distance reduction, if this is employed. The available pencil beam transversal widths (spot sizes) are a function of the beam energy [13]. At our facility, for proton beam energies in the range needed to treat head tumors (50–220 MeV), the smallest available spot sizes in air, expressed as the full width half maximum (FWHM) range for the highest and lowest energy respectively, range from 11 to 32 mm at d_ISO_ and from 8 to 22 mm at d_ISO-40_. These spot sizes were used in combination with a raster pitch of 3 mm and in-depth Bragg peak spacing of 3 mm. No passive devices (ripple filter or other absorbers) were used. An additional tolerance, expressed as a 3D margin, was set, allowing the TPS to place pencil beams outside the PTV, to ensure target coverage and counteract high fluence spots on its border. This margin was adjusted individually for each patient, based on experience with the specific TPS version, to values ranging 4-5 mm laterally, 2–3 mm proximally and 3–4 mm distally.

### Evaluation

PTV coverage and dose conformity were assessed through several dosimetric indexes: near maximum and near minimum dose (D_near-max_, D_near-min_) [14], mean dose (D_mean_) and conformity index (CI) [15].

To assess the integral dose and OAR sparing, both plans of each patient were compared in terms of dose distributions, dose volume histograms (DVH) and selected dosimetric indexes: D_near-min_, D_near-max_ [14], D_mean_ and the volume percentage receiving at least 10% (V_10%_), 60% (V_60%_), 80% (V_80%_) or 95% (V_95%_) of the prescribed dose.

### Statistical analysis and evaluation

For statistical comparisons a nonparametric paired-sample Wilcoxon signed-rank test, with a significance level of 0.05, was used. The calculations were performed with the R statistical package [16].

## Results

### Target volume coverage

PTV coverage of all plans was adequate and consistent with the ICRU guidelines [14]. Additionally, no significant differences were observed in D_near-min_, D_near-max_, D_mean_ and CI_95%_ between the plans, regardless of the distance to the nozzle, as presented in Table 2.

**Table 2:** Planning target volume coverage for treatment plans prepared in two different room positions

**Dosimetric index**	**d**_**ISO**_	**d**_**ISO-40**_	***p***
D_min_ [Gy(RBE)]	46.4 (44.9÷47.6)	46.0 (43.9÷47.1)	0.791
D_near-min_ [Gy(RBE)]	48.5 (48.4÷48.9)	48.6 (48.5÷48.9)	0.068
D_mean_ [Gy(RBE)]	50.4 (50.3÷50.5)	50.4 (50.2÷50.5)	0.204
D_near-max_ [Gy(RBE)]	52.2 (51.8÷52.6)	52.1 (51.7÷52.3)	0.021*
D_max_ [Gy(RBE)]	53.8 (53.1÷55.7)	54.3 (53.1÷55.4)	0.126
CI_95%_ [−]	0.69 (0.45÷0.81)	0.70 (0.49÷0.83)	0.042*
CI_50%_ [−]	0.30 (0.19-0.41)	0.36 (0.22-0.47)	0.002*

Values are median (range) of patient cohort.

*Abbreviations: RBE* relative biological effectiveness, *D*_*(near-)min*_ (near) minimum dose, *D*_*(near-)max*_ (near) maximum dose, *D*_*mean*_ mean dose, *CI*_*X%*_ conformity index of PTV coverage with X% of prescription dose, *d*_*ISO*_ nozzle-to-treatment-isocenter 100 cm (treatment at room isocenter), *d*_*ISO-40*_ nozzle-to-treatment-isocenter 60 cm.

* statistically significant difference (p < 0.05 two-tailed Wilcoxon signed-rank test).

### Normal tissue and OAR sparing

**Figure 2:**
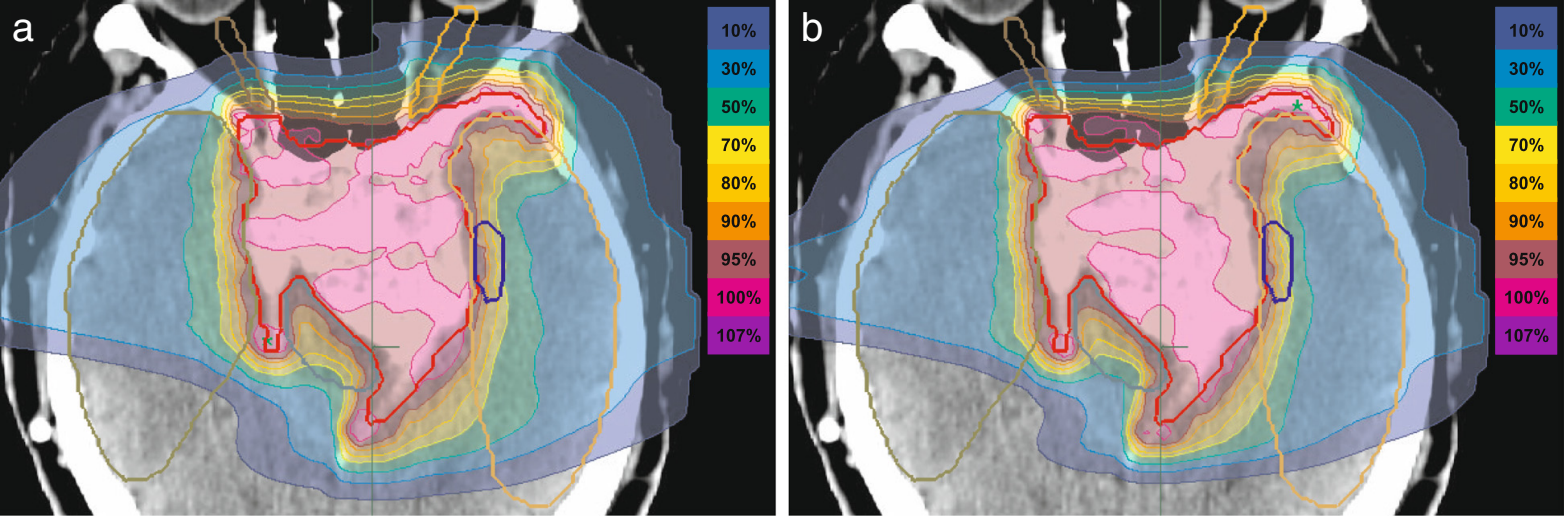
**Dose distribution in a representative axial CT slice of a selected patient for the IMPT plans created using two distances between nozzle and treatment isocenter: a) 100 cm (d**_**ISO**_**) and b) 60 cm (d**_**ISO-40**_**).** The reduced distance between the nozzle and patient leads to a sharper lateral dose fall-off, hence to improved target conformation.

**Figure 3:**
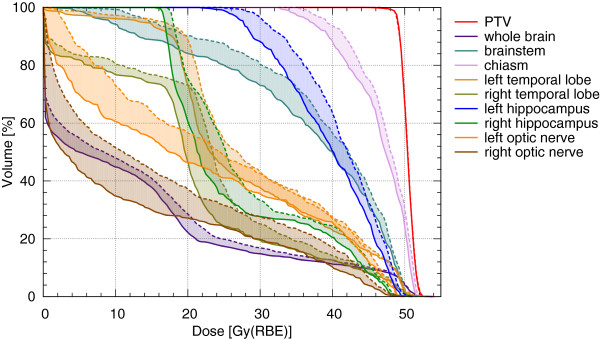
**Dose-volume histograms of selected OARs of an exemplary case for both IMPT plans: nozzle-to-patient distance 100 cm (d**_**ISO **_**- dashed line) and nozzle-to-patient distance 60 cm (d**_**ISO-40 **_**- solid line).**

Figure 2 and Figure 3 illustrate in an exemplary case that sparing of normal tissues was more effective at the treatment position closer to the nozzle. Dose-volume comparisons for the whole brain and selected OARs for all 12 patients are shown in Table 3. In all cases, improved sparing of OARs was achieved at the treatment isocenter position closer to the nozzle (d_ISO-40_). Statistically significant reduction of the integral dose and substantial sparing of the OARs in terms of D_mean,_ V_2%,_ V_10%,_ V_60%_ and V_80%_ were achieved in the whole patient cohort owing to the decreased pencil beam widths.

**Table 3:** Normal brain tissue sparing dose-volume comparisons

**Volume of interest/dosimetric index**	**Absolute difference**		**Relative percentage difference**		***p***
Whole brain					
D_near-max_ [Gy(RBE)]	**0.1**	(−0.4÷3.0)	**0.3**	(−0.7÷8.5)	0.013*
D_mean_ [Gy(RBE)]	**1.0**	(0.5÷1.6)	**12.5**	(9.2÷16.0)	0.001*
V_1%_ [%]	**6.9**	(4.1÷8.6)	**16.7**	(10.4÷21.0)	<0.001*
V_2%_ [%]	**6.3**	(3.4÷7.6)	**15.2**	(10.2÷19.9)	<0.001*
V_10%_ [%]	**4.0**	(2.4÷5.0)	**13.6**	(8.0÷18.4)	<0.001*
V_60%_ [%]	**1.1**	(0.2÷2.0)	**11.3**	(6.4÷16.4)	<0.001*
V_80%_ [%]	**0.4**	(0.1÷1.2)	**6.3**	(2.0÷12.1)	<0.001*
V_95%_ [%]	**0.0**	(−0.3÷0.3)	**1.3**	(−3.8÷7.0)	0.212
Brainstem					
D_near-max_ [Gy(RBE)]	**0.0**	(−0.2÷1.1)	**0.0**	(−0.4÷2.1)	0.173
D_mean_ [Gy(RBE)]	**2.9**	(1.0÷5.0)	**16.2**	(2.6÷30.9)	<0.001*
V_2%_ [%]	**9.6**	(0.0÷22.5)	**14.0**	(0.0÷27.0)	0.002*
V_10%_ [%]	**7.9**	(0.6÷20.1)	**14.8**	(0.6÷31.1)	<0.001*
V_60%_ [%]	**5.5**	(2.0÷8.8)	**17.6**	(3.8÷34.4)	<0.001*
V_80%_ [%]	**2.5**	(0.3÷6.7)	**17.2**	(0.5÷40.0)	<0.001*
V_95%_ [%]	**0.6**	(−0.6÷2.7)	**9.1**	(−14.4÷30.7)	0.017 *
Chiasm					
D_near-max_ [Gy(RBE)]	**0.0**	(−1.5÷4.07)	**0.0**	(−2.9÷9.9)	0.695
D_mean_ [Gy(RBE)]	**0.1**	(−1.2÷4.7)	**0.2**	(−2.5÷26.4)	0.077
V_2%_ [%]	**0.0**	(0.0÷14.6)	**0.0**	(0.0÷15.8)	0.5
V_10%_ [%]	**0.0**	(0.0÷13.6)	**0.0**	(0.0÷17.7)	0.185
V_60%_ [%]	**0.0**	(0.0÷13.3)	**0.0**	(0.0÷59.9)	0.050
V_80%_ [%]	**0.0**	(0.0÷7.0)	**0.0**	(0.0÷64.5)	0.030*
V_95%_ [%]	**0.0**	(−5.1÷8.7)	**0.0**	(−158.5÷26.7)	0.147
Left temporal lobe					
D_near-max_ [Gy(RBE)]	**1.1**	(−0.1÷5.3)	**2.7**	(−0.2÷18.0)	0.001*
D_mean_ [Gy(RBE)]	**1.3**	(0.5÷2.7)	**9.7**	(5.8÷27.4)	<0.001*
V_2%_ [%]	**6.2**	(0.8÷15.4)	**8.8**	(0.8÷38.2)	<0.001*
V_10%_ [%]	**4.7**	(1.4÷9.7)	**7.2**	(1.5÷40.0)	<0.001*
V_60%_ [%]	**1.2**	(0.0÷5.5)	**13.1**	(0.0÷63.9)	0.002*
V_80%_ [%]	**0.4**	(−0.0÷4.0)	**11.3**	(−2.0÷33.3)	0.003*
V_95%_ [%]	**0.0**	(−1.4÷1.2)	**5.7**	(−28.9÷50.0)	0.380
Right temporal lobe					
D_near-max_ [Gy(RBE)]	**0.6**	(−0.2÷2.7)	**1.9**	(−0.4÷7.9)	0.003*
D_mean_ [Gy(RBE)]	**2.3**	(0.5÷3.0)	**15.4**	(7.0÷28.7)	<0.001*
V_2%_ [%]	**7.1**	(2.4÷16.5)	**12.1**	(2.5÷38.0)	<0.001*
V_10%_ [%]	**4.5**	(1.7÷12.9)	**11.5**	(2.0÷36.4)	<0.001*
V_60%_ [%]	**2.7**	(0.0÷6.9)	**20.7**	(0.0÷28.2)	0.002*
V_80%_ [%]	**0.6**	(−0.0÷2.3)	**10.2**	(−4.8÷18.3)	0.003*
V_95%_ [%]	**0.0**	(−0.8÷2.0)	**0.0**	(−10.3÷63.9)	0.207
Left hippocampus					
D_near-max_ [Gy(RBE)]	**0.3**	(−0.1÷4.4)	**0.7**	(−0.3÷76.6)	0.002*
D_mean_ [Gy(RBE)]	**1.9**	(0.3÷5.3)	**10.0**	(0.8÷81.8)	0.001*
V_2%_ [%]	**0.9**	(0.0÷15.0)	**0.9**	(0.0÷85.4)	0.007*
V_10%_ [%]	**5.8**	(0.0 ÷18.5)	**6.6**	(0.0÷93.6)	0.005*
V_60%_ [%]	**1.7**	(−0.2÷12.0)	**10.1**	(−0.2÷21.7)	0.007*
V_80%_ [%]	**0.7**	(0.0 ÷13.1)	**9.9**	(0.0÷42.8)	0.007*
V_95%_ [%]	**0.0**	(−0.3÷5.0)	**0.9**	(−3.7÷44.7)	0.040*
Right hippocampus					
D_near-max_ [Gy(RBE)]	**0.5**	(0.0÷2.3)	**1.0**	(0.0÷100.0)	<0.001*
D_mean_ [Gy(RBE)]	**2.5**	(0.1÷4.2)	**12.9**	(4.9÷100.0)	<0.001*
V_2%_ [%]	**1.6**	(0.0÷19.4)	**2.4**	(0.0÷98.1)	0.007*
V_10%_ [%]	**7.1**	(0.0÷14.9)	**9.5**	(0.0÷19.0)	0.007*
V_60%_ [%]	**5.9**	(−0.2÷22.1)	**15.3**	(−184.6÷42.4)	0.004*
V_80%_ [%]	**2.6**	(−0.0÷9.1)	**11.7**	(−16.7÷75.3)	0.004*
V_95%_ [%]	**0.1**	(−0.5÷7.5)	**0.8**	(−14.3÷62.3)	0.118
Left optic nerve					
D_near-max_ [Gy(RBE)]	**0.1**	(−0.9÷3.9)	**0.2**	(−2.0÷100.0)	0.153
D_mean_ [Gy(RBE)]	**2.2**	(−0.3÷4.0)	**11.8**	(−0.7÷100.0)	0.004*
V_2%_ [%]	**7.3**	(0.0÷17.7)	**11.0**	(0.0÷59.4)	0.005*
V_10%_ [%]	**8.4**	(0.0÷16.8)	**13.7**	(0.0÷49.1)	0.005*
V_60%_ [%]	**2.0**	(0.0÷7.1)	**5.9**	(0.0÷26.1)	0.003*
V_80%_ [%]	**0.9**	(−2.1÷4.7)	**2.8**	(−41.6÷20.0)	0.042*
V_95%_ [%]	**0.2**	(−11.0÷2.1)	**2.1**	(−42.9 ÷40.6)	0.172
Right optic nerve					
D_near-max_ [Gy(RBE)]	**0.1**	(−0.6÷7.3)	**0.3**	(−1.2 ÷97.8)	0.050
D_mean_ [Gy(RBE)]	**2.5**	(0.0÷5.1)	**12.5**	(0.0÷100.0)	0.002*
V_2%_ [%]	**10.8**	(0.0÷25.7)	**14.3**	(0.0÷56.7)	0.002*
V_10%_ [%]	**9.0**	(0.0÷18.2)	**13.2**	(0.0÷38.1)	0.003*
V_60%_ [%]	**3.3**	(0.0÷7.8)	**11.6**	(0.0÷48.3)	0.003*
V_80%_ [%]	**1.3**	(0.0÷7.1)	**8.4**	(0.0÷42.4)	0.003*
V_95%_ [%]	**0.2**	(−1.2÷3.4)	**2.4**	(−57.4 ÷52.3)	0.078

Values are median (range) of patient cohort.

*Abbreviations: RBE* relative biological effectiveness, *D*_*near-max*_, near maximum dose, *D*_*mean*_ mean dose, *V*_*X%*_ percentage of the volume receiving at least X % of the prescribed dose.

* statistically significant difference (p < 0.05 one-tailed Wilcoxon signed-rank test).

The median reduction of D_mean_ for a treatment closer to the nozzle, as compared to a treatment at the room isocenter, was 12.5% to the whole brain, 16.2% to the brainstem, 9.7% to the left temporal lobe, 15.4% to the right temporal lobe, 10.0% to the left hippocampus, 12.9% to the right hippocampus, 0.2% to the optic chiasm, 11.8% to the left optic nerve and 12.5% to the right optic nerve. The reduction of D_mean_ was statistically significant for all organs at risk (*p ≤* 0.004), except the optic chiasm.

The reduced distance between nozzle and patient, resulted in a median V_2%_ decrease by 15.2% for the whole brain, 14.0% for the brainstem, 8.8% for the left temporal lobe, 12.1% for the right temporal lobe, 0.9% for the left hippocampus, 2.4% for the right hippocampus, 11.0% for the left optic nerve, 14.3% for the right optic nerve, while no reduction was observed for the optic chiasm. All V_2%_ reductions in OARs were statistically significant (*p* ≤ 0.007). Also the V_10%_, V_60%_ and V_80%_ were significantly reduced for all OARs (respectively *p ≤* 0.007, *p ≤* 0.007 and *p ≤* 0.042), except the optic chiasm (Table 3).

The maximum dose to OAR remained similar and with statistically significant(*p* < 0.05) tissue sparing at the reduced nozzle-to-patient distance in selected OARs only, clearly because of the very close proximity, if not direct contact, of most OARs with the PTV. Similarly, a reduction of D_near-max_ at the reduced nozzle distance was observed only in a minority of cases and reached statistical significance for selected OARs only: whole brain (*p* = 0.013), left temporal lobe (*p* = 0.001), right temporal lobe (*p* = 0.003), left hippocampus (*p* = 0.002) and right hippocampus (p < 0.001).

Normal tissue sparing achieved with treatment at the reduced distance between nozzle and patient varied between cases as reflected by the considerable range of differences (Table 3).

## Discussion

The main rationale for preferring proton radiotherapy over modern photon techniques in selected clinical situations is the highly conformal dose distribution, with steep dose gradients, enabling not only homogeneous target coverage, but also excellent sparing of OARs and, consecutively, reduced risk of normal tissue complications. To avoid deterioration of these excellent dose localization properties of protons, in the particular treatment room geometry of combined proton-carbon raster-scanning treatment facilities, technical means should be exploited to counteract beam broadening caused by proton scatter. Consequently, we investigated the advantage of beam delivery at reference points, which are closer to the nozzle than the room isocenter. Rather than modeling the effects in simple test target geometries, attention was turned to the dosimetric consequences in a realistic clinical set up.

The reduced nozzle-to-patient distance enabled treatment planning with sharper lateral penumbra and resulted in improvement of the dose conformity for all patients included in this study, as reflected in values of the PTV CI_50%_ conformity index and in the moderate, but statistically significant, sparing of adjacent normal structures typically affecting the D_mean_ while preserving the D_max_, and in the reduction of the integral dose.

Improvements in dose conformity in individual patients, by the reduced nozzle-to treatment isocenter distance, depend on many factors like number of beams, individual patient geometry and the degrees of freedom in the beam setup. The steepness of the dose gradient can be enhanced by a reduced nozzle to patient distance. Therefore, in geometries, where critical organs at risk are located in close proximity of the beam path and directly adjacent to the target, a relevant reduction of the maximum dose to such OAR cannot be expected. Volumes receiving lower doses can however be reduced, partially to an extent, which might lead to a clinical benefit. The critical structures typically accounted for during the treatment planning for cranial lesions, namely brainstem, optic nerves and optic chiasm, due to their relatively well documented morbidity probabilities and established tolerance doses [17]. In the cohort of patients examined in our study, the optic apparatus was typically abutting the PTV and therefore, sharpening of the beam penumbra, reduced the mean dose (D_mean_) in the optic apparatus but in most of the cases did not influence the maximal dose (D_max_). Only in two patients the D_max_ recorded in the nervi optici was appreciably reduced. In these cases, the considered structures were located laterally to the PTV, in close vicinity but not adjacent to it.

In the brainstem, evidence exists concerning dose-volume effects. Using conventional fractionation of 2 Gy/fraction, the entire brainstem may be treated to 54 Gy with limited risk of severe neurologic effects, while smaller volumes (<10 cm^3^) may be irradiated to maximum doses of 59 Gy [18]. Reduction of the penumbra width might therefore be of the advantage as for high-dose treatments more complex constraints on the brainstem are used, allowing higher dose in the brainstem surface but employing stronger restriction for the central part [18].

With improving conformity of new radiation techniques resulting in enhanced tumor control and hence prolonged survival, more data on late cognitive toxicities became available. This as well as the possibility of better sparing of adjacent healthy tissue unmatched by the conventional treatment modalities put new aspects of the quality-of-life of the long-term cancer survivors in the focus resulting in definition of additional OARs. This aspect is of major importance in pediatric cases.

Increasing evidence exists, that besides reduction of volumes of normal tissues receiving high doses, limiting medium and low dose exposure has high relevance for prevention of late radiation morbidity [19]. The risk of neurocognitive impairment after cranial radiotherapy strongly depends on dose volume effects, where the dose to brain sub-volumes plays a crucial role. Sparing of temporal lobes as well as hippocampi is of special relevance as preclinical and clinical evidence suggests, that radiation dose received by the neural stem cells of the subgranular zone in the hippocampus plays a role in radiation-induced neurocognitive decline, specifically memory recall especially in the immature brain [20,21]. To date, no specific constraints have been defined for these structures, however some evidence of a dose-volume relationship was observed.

Merchant *et al.*[22] correlated dose-volume data of 5 sub-volumes (total brain, supratentorial brain, infratentorial brain, and left and right temporal lobes) with intelligence quotient (IQ) at follow-up. Exposure to the supratentorial brain appeared to have the most significant impact. Each Gy of exposure had a similar effect on IQ decline, regardless of dose level. These results underline the importance of measures to reduce radiation dose and treatment volume at all dose levels [22]. Blomstrand *et al.*[21] estimated, that a reduction of the mean dose to both hippocampi from 20.7 Gy to 18.0 Gy limits the risks for developing memory impairment from 47% (95% confidence interval, 21-69%), to 44% (95% confidence interval, 21-65%). In our study, the beam directions were chosen to assure best hippocampal sparing by rotating the beams to pass tangentially to the hippocampi whenever possible. Owing to this configuration, the reduction of the mean dose to the hippocampi was in the same range as in the described study, with a median reduction of D_mean_ by 1.9 Gy(RBE) (0.3-5.3 Gy(RBE)) for the left side and by 2.5 Gy(RBE) (0.1-4.2 Gy(RBE)) for the right side. Hence, the reduction of the D_mean_ and other dose-volume parameters (like V_10%_, V_60%_) is expected to be beneficial in minimizing the side effects.

However, for skull-base tumor treatments, especially at fixed-beam facilities, the temporal lobes are almost unavoidably in the beam channels. In this case, the advantage of the patient-to-nozzle distance reduction manifests itself solely in the reduction of the channel cross-section, and therefore the volume of the organ involved and exposed to low and medium doses, especially for larger tumors.

Finally, the D_mean_ as well as V_10%_, V_2%_ and V_1%_ of the whole brain volume were significantly reduced, which could be of special importance in the context of secondary cancer induction as a late effect of radiation treatment. Recent findings suggest, that decreasing the volume receiving low to intermediate doses is decisive to decrease the rate of secondary tumors, [23,24]. In the study by Diallo *et al.*[23], 66% of secondary tumors occurred in the beam bordering region (i.e. the area surrounding the PTV), where normal tissue sparing can be improved, as demonstrated in our study, with a reduced distance between nozzle and treatment isocenter.

In centers equipped with a gantry, a greater effect can be expected by a reduced nozzle-to-patient distance owing to more degrees of freedom in choosing the beam directions. In particular, as due to range uncertainties, in particle therapy planning techniques are preferred, where the critical structures are located at the lateral rather than the distal edge of the beam [25]. This is especially important for cyclotron based facilities with passive energy variation obtained by insertion of range shifters, as it results with larger beam divergence at the nozzle exit. Therefore, the existing literature puts stress on keeping the patient close to the nozzle [25]. At the particle therapy center in Marburg, with the current technical solution, shifting of patients by a maximum of about 40 cm towards the nozzle is possible (the maximum depends on the isocentric angle). In principle, further decreasing of the nozzle-to-patient distance would allow for further reduction of the dose to organs at risk. Additional improvements in conformity might be achieved by adding a non-coplanar field, which is however not preferable due to unnecessary involvement of the normal brain tissue in the beam channel.

It should be noticed that shifting the treatment isocenter closer to the nozzle puts further demands on quality assurance (QA) procedures as mechanical, geometrical and dosimetric (also patient specific QA) tests have to be performed in at the new, shifted reference points. QA procedures for treatment planning and for patient positioning must also be extended for the shifted treatment positions. Especially, the image guided positioning verification requires a careful QA, because in most cases it is not feasible in the shifted treatment positions and therefore implies a “blind” table movement between imaging and irradiation. Laser control allowing for visual patient positioning control as a plausibility check prior irradiation is desirable. Appropriate QA- and workflow procedures must therefore ensure patient safety during daily clinical routine. Technical measures and software assistance, with appropriate interlocks, are necessary to reliably avoid collision of the devices and especially treatment at incorrect positions. All additional QA-requirements are however feasible and technical support tools partly installed.

## Conclusion

A reduced distance between the vacuum window of the nozzle and the treatment isocenter leads to a steeper lateral dose fall-off along the beam path and consequently offers the possibility to further improve dose distribution conformity in the particular geometry of combined proton-carbon ion fixed-nozzle facilities with scanning technology. Clinical benefit can be expected due to a reduction of the volume of irradiated normal tissues, especially of the volume of OARs directly adjacent to the beam channel, which receive low to intermediate doses. The maximum dose to the OARs directly adjacent to the target volume remains unchanged. The benefit for individual patients depends on the individual patient geometry, number of beams and the degrees of freedom in the beam setup. Use of this option should therefore be considered on an individual basis. Shifting of the treatment position closer to the nozzle and out of the room´s isocenter requires appropriate quality assurance procedures to ensure safe and correct treatments.

## Competing interests

The authors declare that they have no competing interests.

## Authors’ contributions

UJ contributed to study design, data acquisition, and physical evaluation of treatment plans, performed the statistical analysis and contributed in writing the final manuscript. MEB performed the treatment planning and drafted the manuscript. FA performed data acquisition and data management and contributed in writing the final manuscript. REC contributed to the study design and revision of the manuscript for the clinical content. UW contributed in conception of the study, reviewing the statistical analysis and revised the manuscript for the technical content. AW contributed to study design, performed patient selection and delineation, medical evaluation of treatment plans and writing of the final manuscript. All authors read and approved the final manuscript.
